# Marine Puupehenone and Puupehedione: Synthesis and Future Perspectives

**DOI:** 10.3390/md21060322

**Published:** 2023-05-26

**Authors:** Antonio Rosales Martínez, Ignacio Rodríguez-García

**Affiliations:** 1 Department of Chemical Engineering, Escuela Politécnica Superior, University of Sevilla, 41011 Sevilla, Spain; 2 Organic Chemistry, ceiA3, CIAIMBITAL, University of Almería, 04120 Almería, Spain; irodrigu@ual.es

**Keywords:** marine natural products, meroterpenoids, puupehenone, puupehedione

## Abstract

Puupehenone and puupehedione are natural products isolated from marine organisms. These compounds display a broad spectrum of biological activities, the in vitro antitubercular activity of puupehenone being a stand out, and are equipped with an interesting structural complexity. These products have served to stimulate the continual interest of the synthetic community. The first part of this article is a review of their total synthesis, using natural compounds which have the potential to be transformed into these marine compounds as starting materials; the synthetic routes employed to generate the basic skeleton; and the advances made to synthesize the pyran C ring with the required diastereoselectivity to obtain the natural products. Finally, this perspective shows a personal reflection of the authors on a possible unified and efficient retrosynthetic route that could allow easy access to these natural products, as well as their epimers at the C8 carbon and which could be used to address future biological issues in the production of pharmacologically active compounds.

## 1. Introduction

**Figure 1:**
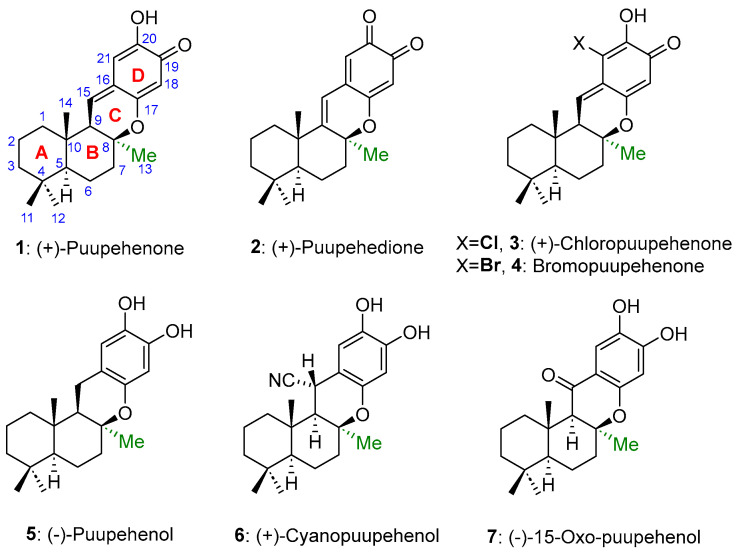
Selected members of puupehenone-type natural compounds (C_8_-Me_α_). (Rings and atoms labels drawn only in **1**).

Quinone/hydroquinone meroterpenoids are constituents of the secondary metabolite extracts of many marine organisms [1]. As a common structural feature, these compounds are constituted by two biogenetically diverse sub-units: a sesquiterpene portion and a quinone or phenolic moiety [2]. Within the meroterpenoid family, there is a remarkable group: the puupehenones. These natural marine compounds have been isolated from sponges, mainly from the orders *Dictyoceratida* and *Verongida*, although some products from this family have been isolated as well from the orders *Haplosclerida* and *Dendroceratida* [3,4,5,6,7,8]. The most representative compounds of this group of marine natural products are (+)-puupehenone (**1**), (+)-puupehedione (**2**), the halopuupehenones (**3**) and (**4**), (-)-puupehenol (**5**), (+)-cyanopuupehenol (**6**), and (-)-15-oxo-puupehenol (**7**) (Figure 1).

Structurally, puupehenone-type natural compounds are tetracyclic products which consist of a *trans*-decalin system (A- and B-rings) and a shikimic acid/O-phenol/O-benzoquinone (D-ring) linked by a tetrahydropyran/dihydropyran (C-ring). The stereochemistry at carbon 8 in this class of natural products is “*S*”, the orientation of the C_8_-Me group being backwards when represented as in Figure 1. This orientation is usually denoted in literature as “α” or C_8_-Me_α_. The opposite orientation, present in some unnatural derivatives, is denoted as “β” or C_8_-Me_β_. The full stereochemistry of (+)-puupehenone (**1**) has been well-established using chemical correlation methods from this compound to finally obtain the known structure of (-)-drimenol [9]. These compounds have great interest for the synthetic and pharmaceutical community due to their unusual fused ring system and their wide variety of biological activities. Natural puupehenones and their C-8 epimers show relevant bioactive properties, exhibiting antitumor, immunomodulatory, antimalarial, antiviral, antibiotic, antiatherosclerotic, and, most remarkably, in vitro antitubercular activity in the case of puupehenone (**1**). Medina and coworkers [10] have published an excellent and complete review of the biological activity of these compounds.

Among all the products that belong to this family, the most representative members are puupehenone (**1**) and puupehedione (**2**), these being the most intensively researched. For this reason, this article highlights the main synthetic efforts toward these marine compounds. Finally, a unified retrosynthesis for the future synthesis of both natural puupehenone-type and non-natural (C_8_-Me_β_) products will be discussed.

## 2. Results

The total synthesis of complex natural compounds remains among the most dynamic areas in organic and pharmaceutical chemistry, and it is an excellent indicator for the validation of new synthetic methodologies. Usually, the starting materials in a total synthesis are commercially available precursors, which are transformed into the natural product of interest through either a convergent synthetic route or a linear synthetic route. In the first case, two fragments are coupled at a late stage to generate the desired natural product, normally through either a nucleophilic or an electrophilic reaction. In a linear synthesis, a series of consecutive transformations (reactions) are employed to transform a reactant or some reactants into a natural product. Normally, this synthetic process entails a longer route to produce the target product.

**Scheme 1:**
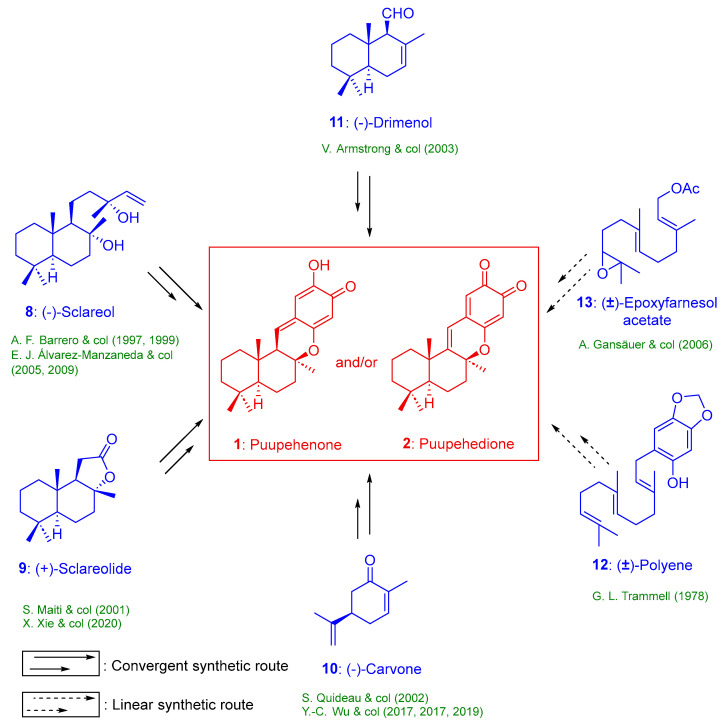
Starting materials employed in the previous synthesis of puupehenone (**1**) and puupehedione (**2**) [11,12,13,14,15,16,17,18,19,20,21,22,23].

Scheme 1 summarizes the pool of chiral starting materials that have been used in the syntheses of puupehenone (**1**) and puupehedione (**2**) through convergent synthetic routes (solid arrows) and the non-chiral starting materials used in the synthesis of **1** and **2** by linear synthetic routes (dashed arrows).

### 2.1. Synthesis of Puupehenone (**1**) and Puupehedione (**2**) from Chiral Starting Materials

#### 2.1.1. Synthesis of Puupehenone (**1**) and Puupehedione (**2**) from (-)-Sclareol (**8**)

(-)-Sclareol (**8**) is a natural bicyclic diterpene alcohol with five stereogenic centers isolated from the herbaceous aromatic plant clary sage (*Salvia sclarea*) [24], a member of the Lamiaceae family [25] native to the Mediterranean basin, Southern Europe, and Iran. The isolation of (-)-sclareol (**8**) requires hydro-distillation of the aerial parts of the plant and is a starting material commercially available, making it an attractive chiral synthon for the asymmetric total synthesis of natural compounds [26].

**Scheme 2:**
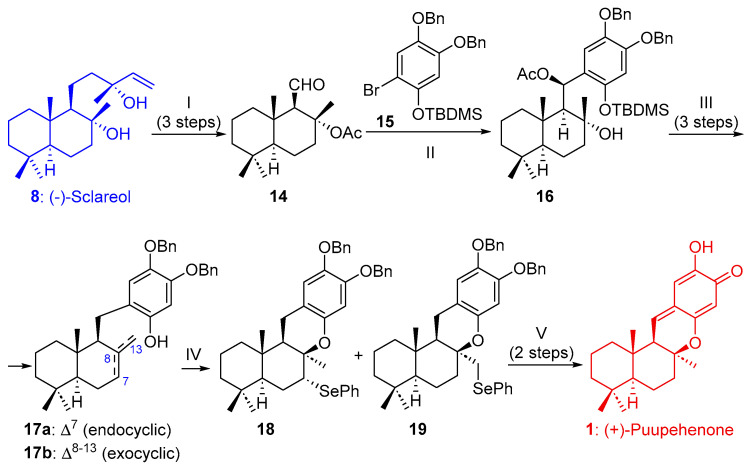
Barrero’s enantioselective synthesis of (+)-puupenhenone (**1**) from (-)-sclareol (**8**). Reagents and conditions: (I) Ref. [27]; (II) **15**, *t*-BuLi, Et_2_O, −78 °C, 88%; (III) (i) Cl_2_SO, Py, rt, 1 h, 94%; (ii) TBAF, THF, rt, 15 min, 81%; (iii) NaBH_4_, EtOH, rt, 20 min, 91%; (IV) NPSP, SnCl_4_, CH_2_Cl_2_, −78 °C, 2 h, 91%; (V) (i) Raney Ni, THF, rt, 20 h, 75%; (ii) PDC, CH_2_Cl_2_, rt, 3 h, 70%. TBAF = tetrabutylammonium fluoride; NPSP = *N*-phenylselenophtalimide; PDC = pyridinium dichromate.

In 1997, Barrero’s group reported the first enantioselective synthesis of marine (+)-puupehenone (**1**) from (-)-sclareol (**8**) and protocatechualdehyde [11] (Scheme 2) via an organoselenium-induced cyclization of a mixture of *endo*/*exo* decalins **17a**–**b** and the simultaneous removal of selenyl groups **18** and **19** using Raney Ni.

The oxidative degradation of commercially available (-)-sclareol (**8**) provided the acetoxy aldehyde **14** (73% yield) in three steps [27]. The nucleophilic addition of the organolithium derived from **15** to the acetoxy aldehyde **14** gave the compound **16** (88% yield) with complete diastereoseletivity. The aromatic bromide ring **15** was obtained from protocatechualdehyde in five steps, with an overall yield of 75%. The compound **16**, after three steps, was converted to phenolic derivatives **17a**–**b** (69% overall yield). Electrophilic cyclization of compounds **17a**–**b** with SnCl_4_ and *N*-phenylselenophtalimide afforded a mixture of selenyl derivatives **18** and **19** (91% yield). Finally, the treatment of **18** and **19** with Raney Ni and subsequent oxidation with pyridinium dichromate gave natural (+)-puupehenone (**1**) (52% yield, two steps). The use of organoselenium-mediated cyclization of the mixture of endocyclic and exocyclic alkenes **17a** and **17b** followed by reduction is an excellent methodology for the synthesis of natural puupehenone-type compounds since, under acid conditions, the hydroxyl group of the aromatic ring in both **17a** and **17b** attacks the double bond of the decalin through the less hindered *α* side, generating the unnatural epimer C_8_-Me_β_ as the main product.

**Scheme 3:**
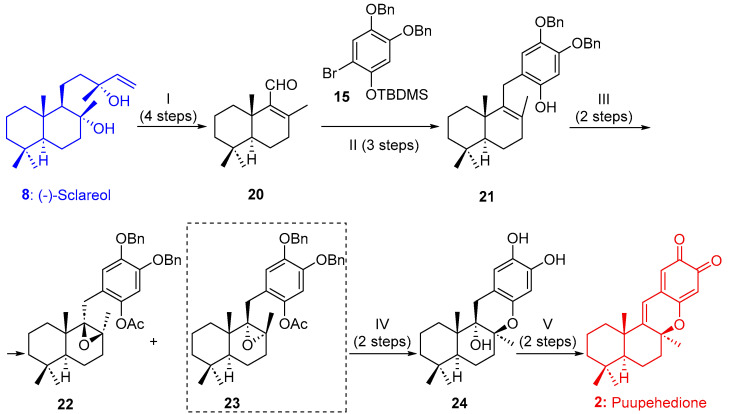
Barrero’s enantioselective synthesis of puupehedione (**2**) from (-)-sclareol (**8**). Reagents and conditions: (I) Ref. [28]; (II) (i) *t*-BuLi, **15**, Et_2_O, −78 °C, 50 min, Ar; **20** Et_2_O, −78 °C, 40 min; (ii) Et_3_SiH, TFA, CH_2_Cl_2_, −78 °C, 45 min, 79%, two steps; (iii) TBAF, THF, rt, 15 min, 95%; (III) (i) Ac_2_O, NaAcO, reflux, 2 h, 98%; (ii) *m*CPBA, NaHCO_3_, CH_2_Cl_2_, −20 °C, 2 h, 98%; (IV) (i) 2N KOH-MeOH, rt, 48 h, 95%; (ii) H_2_, 10% Pd/C, MeOH, rt, 1 h, 91%; (V) (i) silica gel, 45%; (ii) NaIO_4_, EtOH-H_2_O, rt, 15 min, 80%. TFA = trifluoroacetic acid; TBAF = tetrabutylammonium fluoride; *m*CPBA = *meta*-chloroperbenzoic acid.

Later, the same research group reported the first enantioselective synthesis of marine puupehedione (**2**) from (-)-sclareol (**8**), using different strategies [12] (Scheme 3). Among the different methodologies investigated, the methodology shown in Scheme 3 gave the highest levels of stereoselectivity. In this strategy, the key step involves a base-mediated cyclization of epoxide **23**. The key merosesquiterpenic intermediate **21** was generated by condensation of the aldehyde **20** with the aromatic compound **15**, subsequent cationic deoxygenation of the resulting alcohol, and final removal of the silyl protecting group. The preparation of the drimanic aldehyde **20** had been previously reported by the same research group from (-)-sclareol (**8**) [28]. As in the method described in Scheme 2, the aromatic derivative **15** was obtained here from protocatechualdehyde. Acetylation of the phenolic product **21** with Ac_2_O and subsequent treatment with *m*-chloroperbenzoic acid gave a mixture of epoxides **22** and **23** (98% yield, 1:1 mixture). After separation of **23** by column chromatography, this compound was treated with KOH in methanol, generating a tetracyclic compound, which, after deprotection of the benzylether groups with palladium on carbon, afforded the diphenol **24** (86% two steps). Finally, the dehydration of **24** and oxidation of the aromatic ring yielded the desired marine puupehedione (**2**).

**Scheme 4:**
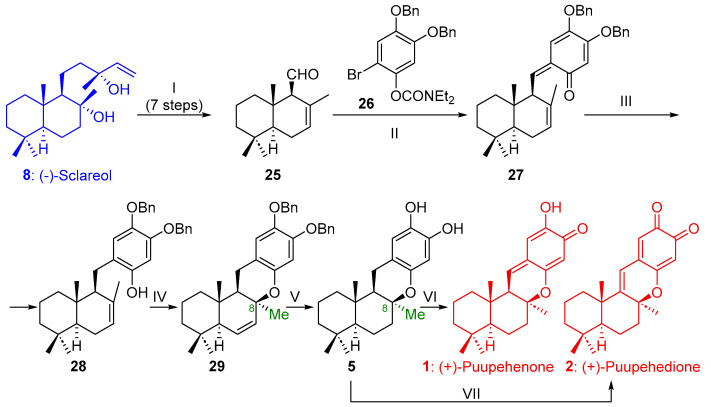
Álvarez-Manzaneda’s enantioselective synthesis of (+)-puupehenone (**1**) and (+)-puupehedione (**2**) from (-)-sclareol (**8**). Reagents and conditions: (I) Ref. [13]; (II) *t*-BuLi, **26**, THF, −80 °C, 30 min, Ar; **25**, THF, −80 °C, 30 min, 87%; (III) Raney Ni, THF, rt, 30 min, 98%; (IV) PdCl_2_, Pd(OAc)_2_ (cat), MeOH-H_2_O (99:1), 40 °C, 48 h, 91%, (V) H_2_, Pd/C, EtOH, rt, 48%, 99%; (VI) Ag_2_O, THF, rt, 90 min, 95%; (VII) DDQ, dioxane, reflux, 2 h, 80%. DDQ = 2,3-Dichloro-5,6-dicyano-1,4-benzoquinone.

In 2005, Álvarez-Manzaneda and co-workers reported a new route toward puupehenone-type marine compounds from (-)-sclareol (**8**) [13] (Scheme 4). The key step was based on the palladium(II)-promoted diastereoselective cyclization of a drimenyl phenol **28**. The drimane skeleton **25** was obtained in seven steps from (-)-sclareol (**8**). The nucleophilic addition of the organolithium derived from **26** to drimenol **25** resulted in a C-C bond formation reaction with concomitant carbamate elimination giving the compound **27**. Bromide aromatic ring **26** was synthesized from 3,4-bis(benzyloxy)phenol in two steps (83% overall yield). Reduction of **27** with Raney Ni afforded **28**. Subsequent cyclization of the drimenyl phenol **28** with catalytic PdCl_2_ and Pd(OAc)_2_ gave the desired compound **29** with complete diastereoselectivity. The catalytic hydrogenation of **29** gave puupehenol (**5**), which is the key compound for the synthesis of puupehenone-type natural products, such as (+)-puupehenone (**1**) and (+)-puupehedione (**2**). Again, to avoid the formation of an unnatural epimer C_8_-Me_β_, the palladium-promoted cycloisomerization strategy turned out to be satisfactory.

**Scheme 5:**
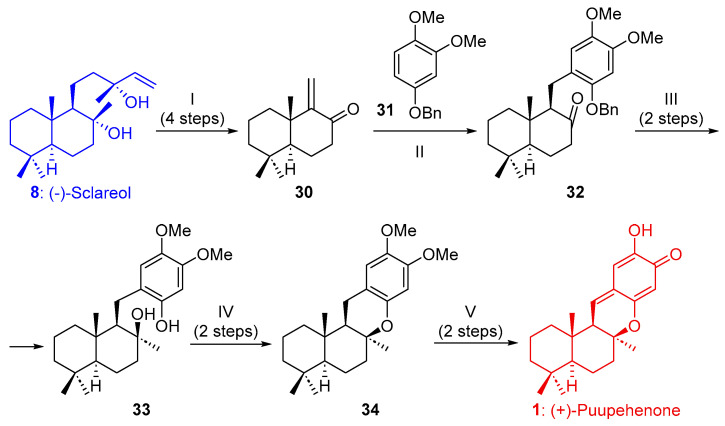
Álvarez-Manzaneda’s enantioselective formal synthesis of (+)-puupehenone (**1**) from (-)-sclareol (**8**). Reagents and conditions: (I) Ref. [14]; (II) Amberlyst A-15, 4 Å molecular sieves, CH_2_Cl_2_, reflux, 12 h, 94%; (III) (i) MeMgBr, Et_2_O, 0 °C, 30 min, 94%; (ii) H_2_, Pd/C, MeOH, rt, 1 h, 93%; (IV) (i) (TfO)_2_O, CH_2_Cl_2_, *i*PrNEt_2_, 0 °C, 5 min, 83%; (ii) Pd(OAc)_2_ (cat), DPPF, NaO*t*Bu, toluene, 100 °C, 14 h, 76%; (V) (i) BBr_3_, CH_2_Cl_2_, 0 °C, 80 min, 82%; (ii) Ref. [13]. DPPF = 1,1′-bis(diphenylphosphanyl)ferrocene.

A different strategy towards puupehenone-type natural products, also using (-)-sclareol (**8**) as a starting material, was reported by the same group [14] (Scheme 5). Here, the key step was based on the cationic-resin-mediated Friedel–Crafts alkylation of the alkoxyarene **31** with the α,β-unsaturated ketone **30**. In turn, the compound **30** had been previously prepared from (-)-sclareol (**8**) in four steps. Treatment of enone **30** with the aromatic ring **31** under a cationic resin Amberslyst A-15 gave the key ketone **32** with complete diastereoselectivity (94% yield).

Treatment of ketone **32** with MeMgBr and further cleavage of the benzyl ether afforded hydroxyphenol **33**. Triflate derivatization of the phenol group in **33** followed by cyclization in the presence of Pd(OAc)_2_, 1,1′-bis(diphenylphosphanyl)ferrocene and sodium *tert*-butoxide, led to the formation of the tetracyclic compound **34**. Finally, deprotection of both methoxy groups resulted in diphenol **5**, previously presented in Scheme 4, which is an advanced intermediate in the Álvarez-Manzaneda’s synthesis of (+)-puupehenone (**1**) [13].

#### 2.1.2. Synthesis of Puupehenone (**1**) and/or Puupehedione (**2**) from (+)-Sclareolide (**9**)

(+)-Sclareolide (**9**) is a sesquiterpene lactone isolated from different plant sources including *Salvia sclarea*, *Salvia yosgadensis*, and cigar tobacco [29,30]. Due to its commercial availability, as well as the correct chirality for three of the four (+)-puupehenone stereogenic centers, this natural compound has been employed as a starting material for the total synthesis of many terpenoids and their derivatives [26,31,32].

**Scheme 6:**
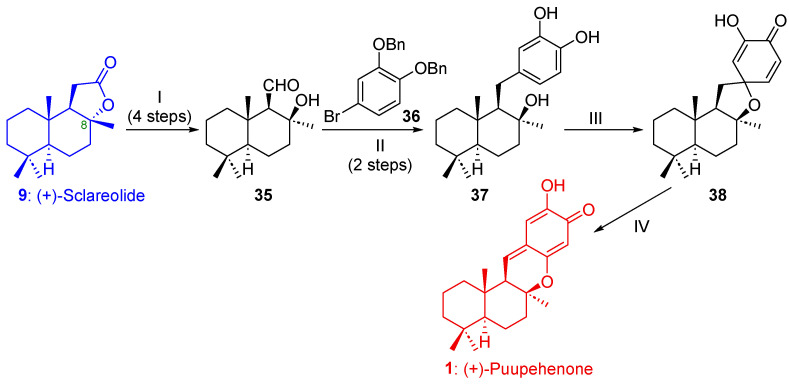
Quideau’s enantiospecific synthesis of (+)-puupehenone (**1**) from (+)-sclareolide (**9**). Reagents and conditions: (I) Ref. [15]; (II) (i) **36**, *t*-BuLi, THF, −78 °C, 20 min; then **35** THF, −78 °C, 15 min, 73%; (ii) H_2_, Pd/C (10%), THF, rt, overnight, 87%; (III) BTI, CH_2_Cl_2_, 45 min, −25 °C, 67%; (IV) KH, dioxane, rt, 1h; then 20 min at 45 °C, 27%. BTI = [bis(trifluoroacetoxy)-iodo]benzene.

Quideau and co-workers were the first research group to describe the use of (+)-sclareolide (**9**) as a starting material for the synthesis of (+)-puupehenone (**1**) [15] (Scheme 6). The key step of this synthesis was based on the nucleophilic character of the terpenoid C8-oxygen of **37** to promote heterocyclization by attacking an oxidatively activated 1,2-dihydroxyphenyl unit. The drimane β-hydroxy aldehyde **35** was synthesized in four steps from (+)-sclareolide (**9**) through a degradative sequence of reactions which included the inversion of the configuration at C8. Then, the C-C bond formation reaction between **35** and the organolithium derived from **36** gave, after deoxygenation and benzyl ether deprotection, the key compound **37**. The aromatic derivative **36** was prepared from cathecol in two steps in a 78% overall yield. Finally, in two reaction steps, (+)-puupehenone (**1**) was obtained from **37**. In this way, BTI oxidation with concomitant cyclization led to the spirocycle **38**, which was rearranged under basic conditions into (+)-puupehenone (**1**).

**Scheme 7:**
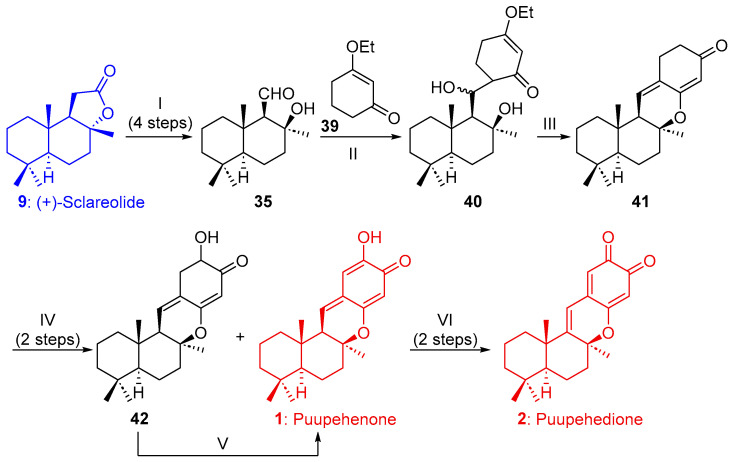
Wu’s enantiospecific synthesis of puupehenone (**1**) and puupehedione (**2**) from (+)-sclareolide (**9**). Reagents and conditions: (I) Ref. [15]; (II) **39**, LDA, THF, −78 °C, 30 min; then **35**, 1h, 67%; (III) HCl, MeOH, rt, 30 min, 92%; (IV) (i) KHDMS, THF, −78 °C, 30 min; (ii) then P(OMe)_3_, O_2_, −78 °C, 1 h, **42** (19%), **1** (38%); (V) *t*BuOK, *t*BuOH rt, 1 h, 86%; (VI) (i) NaBH_4_, EtOH, rt, 20 min, 92%; (ii) DDQ, 1,4-dioxane, reflux, 2h, 71%. DDQ = 2,3-dichloro-5,6-dicyano-1,4-benzoquinone.

More recently, in 2017, Wu et al. [16] reported a green synthesis of puupehenone (**1**) and puupehedione (**2**) which avoided the use of protecting groups and also started from (+)-sclareolide (**9**). In this strategy, the key step is a hemiacetalization/dehydroxylation/hydroxylation/retro-hemiacetalization tandem reaction of **40** (Scheme 7). The drimanic β-hydroxyaldehyde **35** was prepared following Quideau’s procedure from commercially available (+)-sclareolide (**9**) [15]. With the compound **35** in their hands, the key intermediate **40** was obtained in a 67% yield via the treatment of ketone **39** with LDA and a subsequent C–C bond forming reaction with aldehyde **35**. Under controlled acidic conditions, **40** underwent a series of one-pot transformations—hemiacetalization, dihydroxylation, hydroxylation, and retro-hemiacetalization—which led to the formation of enone **41** without C8 epimerization (92% yield). The reaction of enone **41** with KHDMS and subsequent oxidation with O_2_ in the presence of P(OMe)_3_ gave puupehenone (**1**) (38% yield) and the α-hydroxylated compound **42** in a 19% yield. Compound **42** was converted in **1** via treatment with *t*-BuOK (86% yield). Finally, reduction of puupehenone (**1**) and subsequent oxidation with DDQ provided puupehedione (**2**) in a 71% yield.

**Scheme 8:**
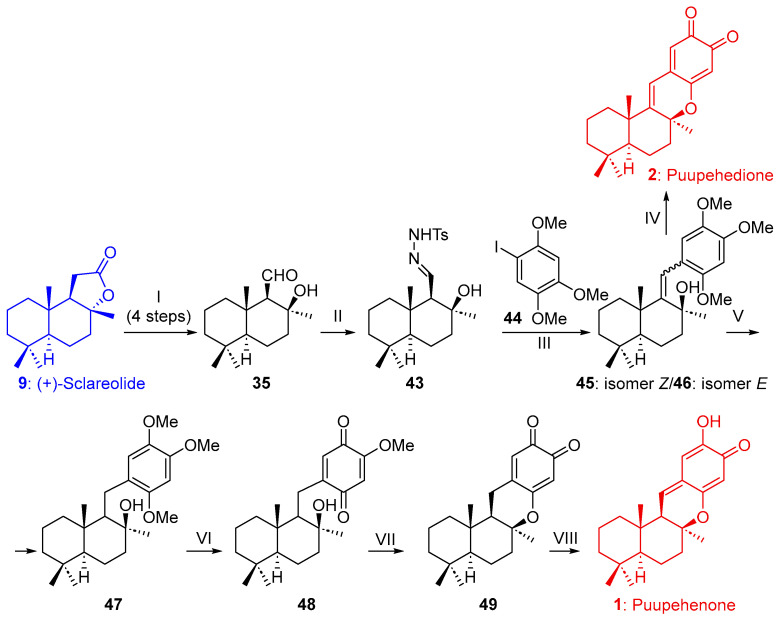
Wu’s enantiospecific synthesis of puupehenone (**1**) and puupehedione (**2**) from (+)-sclareolide (**9**). Reagents and conditions: (I) Ref. [15]; (II) *p*TsNHNH_2_, MeOH, rt, 12 h, 88%; (III) **44**, Pd(PPh_3_)_4_ (cat), K_2_CO_3_, toluene, 110 °C, 8 h, 85%; (IV) CAN, MeCN-H_2_O, rt, 47%; (V) Pd/C, H_2_, MeOH, 40 °C, 62%; (VI) CAN, MeCN-H_2_O, −5 °C to rt, 84%; (VII) *p*TsOH, CH_2_Cl_2_, rt; (VIII) K_2_CO_3_, CH_3_CN, rt, 92% from **48**. CAN = ceric ammonium nitrate.

In connection with the previously mentioned synthetic route, the same research group reported an enantiospecific and concise semisynthesis of puupehenone (**1**) and puupehedione (**2**) using (+)-sclareolide (**9**) as a starting material in only seven steps while also avoiding the use of protecting groups [17] (Scheme 8). The main features of this synthetic route were the development of a palladium-catalyzed C–C bond formation reaction between drimanal hydrazone **43** and the aryliodine **44** for the synthesis of the key intermediate drimanal trimethoxystyrene skeleton **45** and **46** and the development of a CAN-oxidation/intramolecular oxa-Stork–Danheiser transposition tandem reaction of **48** to form the C and D rings of puupehenone (**1**). Again, the β-hydroxyaldehyde **35** was prepared from (+)-sclareolide (**9**) using the procedure previously reported [15]. The condensation of **35** with *p*-toluensulfonyl hydrazide afforded the intermediate **43**. The cross-coupling reaction between hydrazone **43** and aryl iodide **44** using catalytic Pd(PPh_3_)_4_ and K_2_CO_3_ gave the key intermediate drimanal-trimethoxystyrene as a mixture of isomers **45** (40% yield) and **46** (45% yield). Finally, the chemoselective oxidation of both isomers **45** and **46** with CAN provided puupehedione (**2**) (47% yield). In addition, hydrogenolysis of this **45**–**46** mixture with Pd/C produced compound **47** in 62% yield, which was transformed into the *p*-benzoquinone **48** by treatment with ceric ammonium nitrate (84% yield). The intramolecular oxa-Stork–Danheiser reaction of **48** with *p*TsOH gave **49**. Finally, puupehenone (**1**) was obtained by the enolization of **49** with K_2_CO_3_ (92% yield).

#### 2.1.3. Synthesis of (+)-Puupehenone (**1**) and/or (+)-Puupehedione (**2**) from (-)-Carvone (**10**)

(-)-Carvone (**10**) is a monoterpene that occurs at high concentrations (70–80%) in spearmint oil [33]. This natural product is economically available, making it an attractive chiral synthon for the enantiospecific total synthesis of natural compounds [34].

**Scheme 9:**
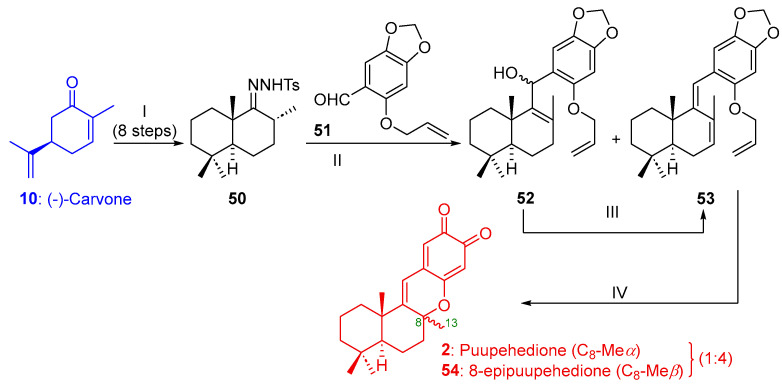
Banerjee’s enantiospecific synthesis of puupehedione (**2**) from (-)-carvone (**10**). Reagents and conditions: (I) Ref. [18]; (II) *n*-BuLi, −78 °C (3 h) to −5 °C (6 h), to rt (2 h); then **50**, −78 °C, 5 h, 59%; (III) silica gel, CH_2_Cl_2_, rt, 3–4 h; (IV) RhCl_3_·3H_2_O, EtOH, reflux, 7 h, quantitative.

In 2001, Banaerjee and coworkers reported an enantiospecific synthesis of puupehedione (**2**) from commercially available (-)-carvone (**10**) [18] (Scheme 9). The key steps were a cross-coupling reaction between a vinyl anion formed from bicyclic tosylhydrazone **50** and the aromatic synthon **51**. Tosylhydrazone **50** was synthesized from natural (-)-carvone (**10**) in eight steps [18], and the aromatic synthon **51** was prepared in three steps with high yield from sesamol [18]. The exposure of the tosylhydrazone **50** to *n*-BuLi formed a vinyl anion, which reacted with **51** to give a mixture of an unstable allyl alcohol **52** and the diene **53**. In fact, **52** suffers dehydration when treated with silica, leading to the diene **53**. Finally, when **53** was treated with RhCl_3_·3H_2_O for *O*-allyl deprotection, an immediate cyclization took place, leading to a mixture of puupehedione (**2**) and its epimer 8-epipuupehedione (C_8_-Me_β_) (**54**) in a ratio 1:4 and in quantitative yield. 8-Epipuupehedione (**54**) was the main compound generated because it is more stable [35] than its natural epimer C_8_-Me_α_ **2**.

**Scheme 10:**
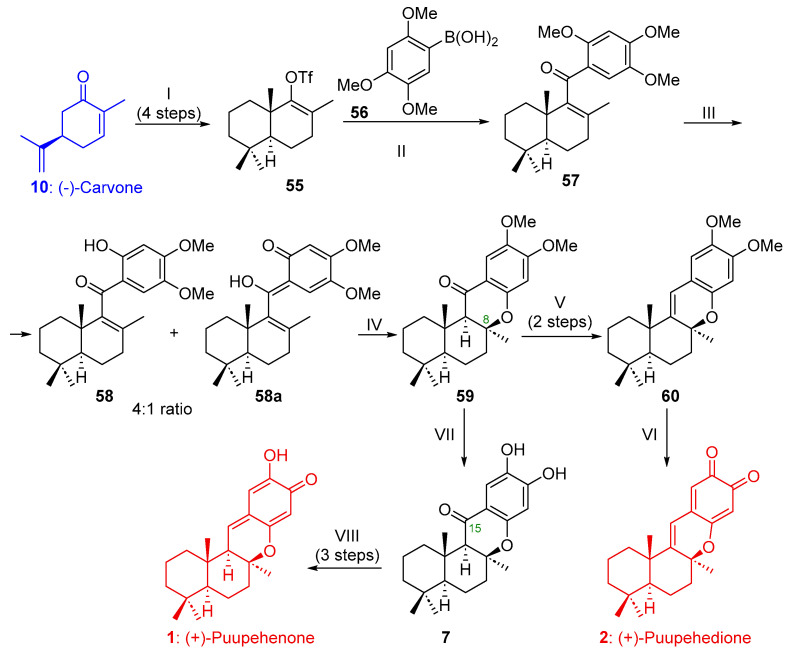
Xie’s enantiospecific and concise synthesis of (+)-puupehenone (**1**) and (+)-puupehedione (**2**) from (-)-carvone (**10**). Reagents and conditions: (I) Ref. [19]; (II) **55**, **56**, PdCl_2_ (cat), PPh_3_, K_2_CO_3_, CO (1 atm), DMF, 80 °C, 2h, rt; then 12 h at 80 °C, 73%; (III) AlCl_3_, CH_3_CN, reflux, 4 h, 90%; (IV) DEG, KOH, 120 °C, 12 h, 86 °C; (V) (i) LiAlH_4_, THF; (ii) TFA/H_2_O = 6/10 equiv, CH_2_Cl_2_, 86% two steps; (VI) Ref. [20]; (VII) BBr_3_, 0 °C, CH_2_Cl_2_, 99%; (VIII) (i) DIPEA, MOMCl, CH_2_Cl_2_, 93%; (ii) LiBH_4_, THF, 0 °C at rt, overnight; (iii) THF/H_2_O: 10/1, *p*-TsOH, 40 °C, 2 h, 56% two steps, based on the recycled starting material. DMF = Dimethylformamide; DEG = Diethylene glycol; TFA = trifluoroacetic acid; DIPEA = *N*,*N*-diisopropylethyl amine; MOMCl = chloromethyl methyl ether.

Using a different approach, but also starting from (-)-carvone (**10**), Xie and coworkers recently reported the total synthesis of (+)-puupehenone (**1**) and (+)-puupehedione (**2**) [19] (Scheme 10). The key steps were a Suzuki carbonylative cross-coupling reaction to combine the bicyclic unit **55** and an aromatic derivative **56** and an intramolecular cyclization reaction to generate the key chromanone core of **59**. The bicyclic vinyl triflate **55** was prepared starting from (-)-carvone (**10**) through a four-step synthetic route [19]. The aromatic derivative **56** was synthesized from commercially available 1,2,4-trimethoxybenzene in two steps following a previously reported procedure [36]. A Suzuki carbonylation cross-coupling reaction between those two segments, **55** and **56**, under CO atmosphere gave the intermediate **57** (73% yield). A subsequent selective demethylation reaction of **57** with AlCl_3_ gave the corresponding demethylated compounds as a 4:1 mixture of tautomers, **58** and **58a** (90% yield). Treatment of this mixture with KOH promoted an intramolecular cyclization, leading to the formation of the compound **59** with the desired stereochemistry at C8 (C_8_-Me_α_) (86% yield). From **59**, the formal synthesis of puupehedione (**2**) and total synthesis of puupehenone (**1**) were completed. Thus, when compound **59** was sequentially treated with LiAlH_4_ and trifluoroacetic acid, the tetracyclic compound **60** was formed (86% yield). As the tetracyclic chromone **60** is an advanced intermediate key in Armstrong et al.’s [20] total synthesis of (+)-puupehedione (**2**), this work constituted a formal synthesis of **2**. On the other hand, the demethylation reaction of compound **59** with BBr_3_ gave (-)-15-oxopuupehenol (**7**). Since a direct reduction of **7** was not successful, the two phenolic groups in **7** had to be protected as the corresponding MOM ethers. Finally, reduction of the C-15 ketone group followed by *p*-TsOH mediated one-pot MOM ether deprotection/dehydration elimination of the C-15 hydroxyl group gave (+)-puupehenone (**1**) in a 56% yield based on the recycled starting material.

#### 2.1.4. Synthesis of Puupehedione (**2**) from (-)-Drimenol (**11**)

(-)-Drimenol (**11**) is a sesquiterpene alcohol which was first isolated from the bark of *Drimys winteri* Forst [37]. This natural compound, which has been employed as a chiral synthon for a large number of natural drimane-type products [38], is typically isolated in a 13% yield from the petroleum ether extract of the bark of the above-mentioned tree.

**Scheme 11:**
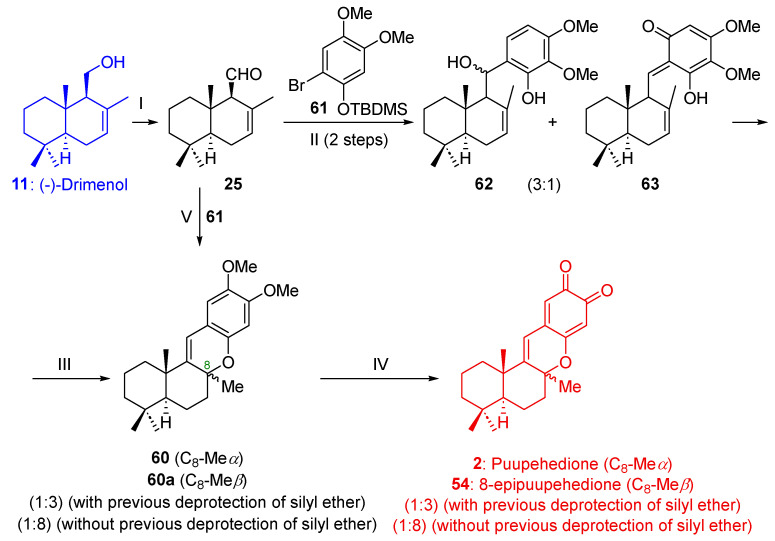
Armstrong’s stereoselective synthesis of puupehedione (**2**) from (-)-drimenol (**11**). Reagents and conditions: (I) PCC, CH_2_Cl_2_ THF (yield was not reported in the original publication); (II) (i) *n*-BuLi, −78 °C, **61**, Et_2_O, **25**; (ii) TBAF, THF; (III) *p*-TsOH, benzene, rt, 16 h, 90% from **25**; (IV) AgO/HNO_3_, THF (yield was not reported in the original publication); (V) *n*-BuLi, **61**, Et_2_O, **25**, *p*-TsOH, benzene. PCC = pyridinium chlorochromate; TBAF = tetrabutylammonium fluoride.

In 2003, Armstrong and co-workers [20] reported the synthesis of puupehedione (**2**) from (-)-drimenol (**11**) (Scheme 11). The key step in this synthetic route was the coupling of drimanal **25** with the aromatic synthon **61**. Drimanal was synthesized from (-)-drimenol (**11**) via oxidation with pyridinium chlorochromate, and the aromatic synthon **61** was prepared from 3,4-dimethoxybenzaldehyde in four steps with a high yield [20]. The addition of drimanal **25** to the aryllithium derived from **61** and subsequent deprotection of the *tert*-butyldimethylsilyl ether gave a mixture of **62** and **63** in a ratio 3:1. The treatment of this mixture with *p*-toluenesulfonic acid gave the cyclization products **60** and **60a** in a 1:3 ratio. Finally, the simultaneous demethylation and oxidation of **60** and **60a** with AgO/HNO_3_ afforded a mixture of puupehedione (**2**) and 8-epipuupehedione (**54**) in the same 1:3 ratio. 8-Epipuupehedione (**54**) was the main compound due to its higher stability. An alternative procedure by direct treatment of the condensation compound with acid, without previous deprotection of the silyl ether (step V, Scheme 11), gave **60** and **60a** in a 1:8 ratio. Simultaneous demethylation and oxidation of **60** and **60a** with AgO/HNO_3_ afforded natural puupehedione (**2**) and its epimer (**54**) in a ratio 1:8. Therefore, this second procedure generated a lower yield of the natural puupehedione (**2**), as it was much more selective towards its unnatural β-epimer.

### 2.2. Synthesis of Puupehenone (**1**) and Puupehedione (**2**) from Non-Chiral Starting Material

#### 2.2.1. Synthesis of (±)-Puupehenone (**2**) from Farnesyl Bromide

**Scheme 12:**
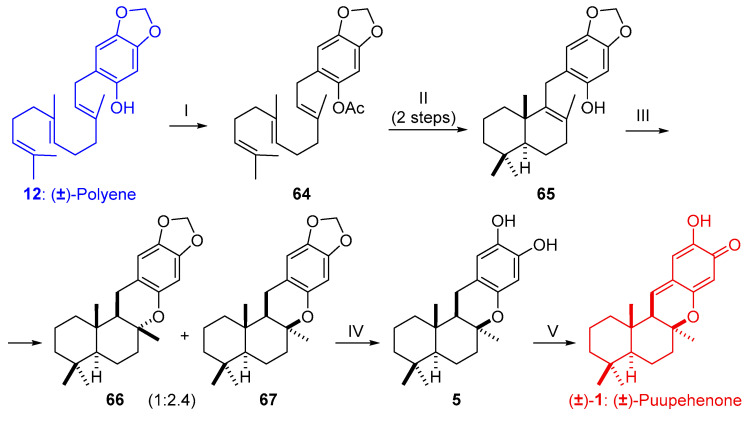
Trammell’s stereoselective synthesis of (±)-puupehenone (**1**) from farnesol bromide. Reagents and conditions: (I) Ac_2_O, pyridine; (II) (i) BF_3_·Et_2_O, CH_2_Cl_2_; (ii) KOH, MeOH, 40% overall yield; (III) β-naphthalenesulfonic acid, CH_2_Cl_2_; (IV) (i) PCl_5_, CH_2_Cl_2_, reflux; (ii) H_2_O, MeOH, reflux; (V) KOH, MeOH, O_2_, quantitative yield.

(*E*,*E*)-Farnesyl bromide, a derivative of natural farnesol, is a commercially available product which has been used in the total synthesis of several natural products [39]. In 1978, Trammell reported the first total synthesis of (±)-puupehenone (**1**) using farnesyl bromide as starting material [21] (Scheme 12). In this procedure a linear synthetic route was used. The key step was an acid-mediated cyclization of the polyene **12**, prepared by alkylation of the lithium salt of sesamol with farnesyl bromide. Cyclization of **12** with boron trifluoride etherate afforded an incompletely cyclized product. For this reason, **12** was acetylated. Cyclization of acetate **64** with BF_3_·Et_2_O followed by alkaline hydrolysis gave phenol **65** (40% overall yield). Subsequent treatment of compound **65** with a catalytic amount of β-naphthalenesulfonic acid afforded a mixture of two isomers, **66** and **67**, in a ratio 1/2.4. The yield of this cyclization was not reported in the original paper. Cleavage of the methylenedioxy protecting group in **67** with phosphorus pentachloride, and subsequent hydrolysis gave (±)-puupehenol **5**. The yield of this deprotection was not reported in the original publication. Finally, aerobic oxidation of phenol **5** in the presence of KOH yielded (±)-puupehenone (**(±)-1**) in an almost quantitative yield. Although Barrero and coworkers later observed the lack of reproducibility of this synthesis [11], the electrophilic addition approach to the oxygen heterocycle probably inspired these researchers, who later reported a second synthesis of puuphenone via an organoselenium-mediated heterocyclization step [11].

#### 2.2.2. Synthesis of (±)-Puupehedione (**(±)2**) from Farnesol

Farnesol is an acyclic sesquiterpene alcohol present in many essential oils [40] such as neroli, citronella, lemon grass, tuberose, cyclamen, musk, tolu, and rose. This natural compound is widely used as starting material in the synthesis of natural products [41,42].

**Scheme 13:**
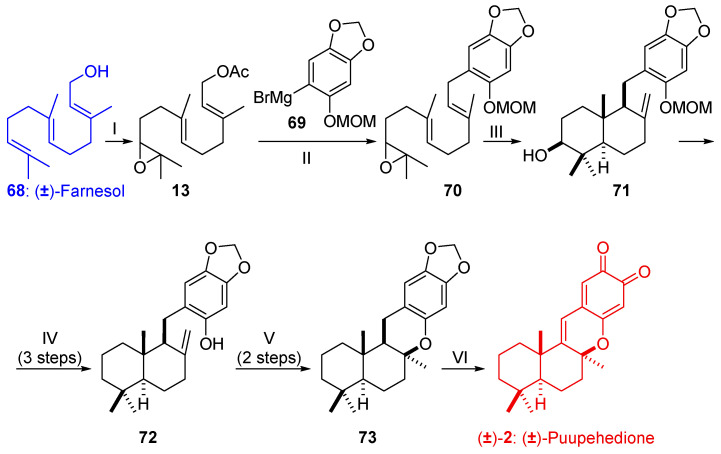
Gansäuer’s diastereoselective synthesis of (±)-puupehedioine (**2**) from farnesol **68**. Reagents and conditions: (I) Ref. [44]; (II) Li_2_CiCl_4_ (cat), **13**, THF, 0 °C; then **69**, 3 h at 0 °C, then overnight at rt, 60%; (III) Cp_2_TiCl (cat), Mn, 2,4,6-collidine, Me_3_SiCl, THF, rt, overnight, 41%; (IV) (i) DMAP, C_6_F_5_OCSCl, CH_2_Cl_2_, 2h, rt, 76%; (ii) HSnBu_3_, AIBN, benzene, reflux, 1 h, 77%; (iii) *p*-TsOH, MeOH, 48 h, rt, 85%; (V) (i) *N*-(phenylseleno)phthalimide, SnCl_4_, CH_2_Cl_2_, 0 °C, 30 min, 80%; (ii) HSnBu_3_, AIBN, benzene, reflux, 1 h, 86%; (VI) Ref. [12]. DMAP = 4-Dimethylaminopyridine; C_6_F_5_OCSCl = *O*-pentafluorophenyl chlorothioformate; AIBN = Azobisisobutyronitrile.

In 2006, Gansäuer et al. [22,43] reported an efficient synthetic approach to (±)-puupehedione (**(±)-2**) from commercially available farnesol (**68**). Some of us collaborated in this synthesis. Key steps in this synthetic linear route were an allylic substitution reaction between **13** and **69**, and the titanocene(III)-catalyzed radical cascade cyclization of a suitable epoxy-polyprene **70** (Scheme 13). The arylepoxypolyene **70** was prepared via the coupling of the epoxyfarnesyl acetate **13** with the benzyl Grignard reagent **69** through an allylic substitution reaction catalyzed by Li_2_CuCl_4_ in a 60% yield. Epoxyfarnesyl acetate **13** was synthesized from farnesol **68**, while benzyl Grignard reagent **69** was obtained using its bromide aromatic ring derivative and magnesium. The *trans*-decaline **71** was prepared by Cp_2_TiCl-catalyzed epoxypolyene diastereoselective cyclization of **70** in 41% yield. The key intermediate **72** was obtained in a sequence of three steps, including deoxygenation of hydroxyl group at C3 via a Barton–McCombie deoxygenation reaction and cleavage of the methoxymethyl ether group of **71** in a 74% overall yield. The reaction of **72** with *N*-(phenylseleno)phthalimide and subsequent reduction with Bu_3_SnH yielded **73** in good overall yield (69%), completing the formal synthesis of marine (±)-puupehedione (**(±)-2**) [12].

Finally, to conclude this section, it is worth indicating that some of the synthetic procedures reported here have enabled the synthesis of other derivatives of puupehenone-type products [23], mainly their epimers at carbon-8, which also turn out to be interesting due to their important biological properties [10].

## 3. Future Perspectives

In light of the exhaustive review of the different syntheses of the marine puupehenone (**1**) and puupehedione (**2**) from accessible natural synthons presented in Section 2, it is obvious that the development of new synthetic methodologies has allowed the efficient construction of the carbon skeletons of these types of compounds. In this way, different synthetic routes to prepare puupehenone (**1**) and puupehedidone (**2**) have been described, including the coupling of the aldehydes with aromatic synthons, the Suzuki carbonylative coupling reaction, the hemiacetalization/dehydroxylation/hydroxylation/retrohemiacetalization tandem reaction, the Friedel–Crafts coupling reaction, and linear synthetic routes. Another important aspect to highlight is the efforts made to achieve high levels of diastereoselection in the formation of the pyran ring of these compounds (C ring) since natural products with their methyl group on C8 with α orientation (C_8_-Me_α_) are less stable than their C_8_-epimers (C_8_-Me_β_). For this purpose, important advances have been made: the use of organoselenium reagents, although with an increase in the number of synthetic operations that must be performed; the palladium(II)-mediated diastereoselective cyclization; the hemiacetalization/dehydroxylation/hydroxylation/retro-hemiacetalization; the diastereoselective intramolecular cyclization, in basic medium, of a skeleton having an oxygen atom attached to C-15 to obtain the desired natural products (C_8_-Me_α_), avoiding the use of protecting groups and transition metals. However, despite all the advances made, we consider that a unified synthesis for the preparation of puupehenone-type compounds is desirable.

**Scheme 14:**
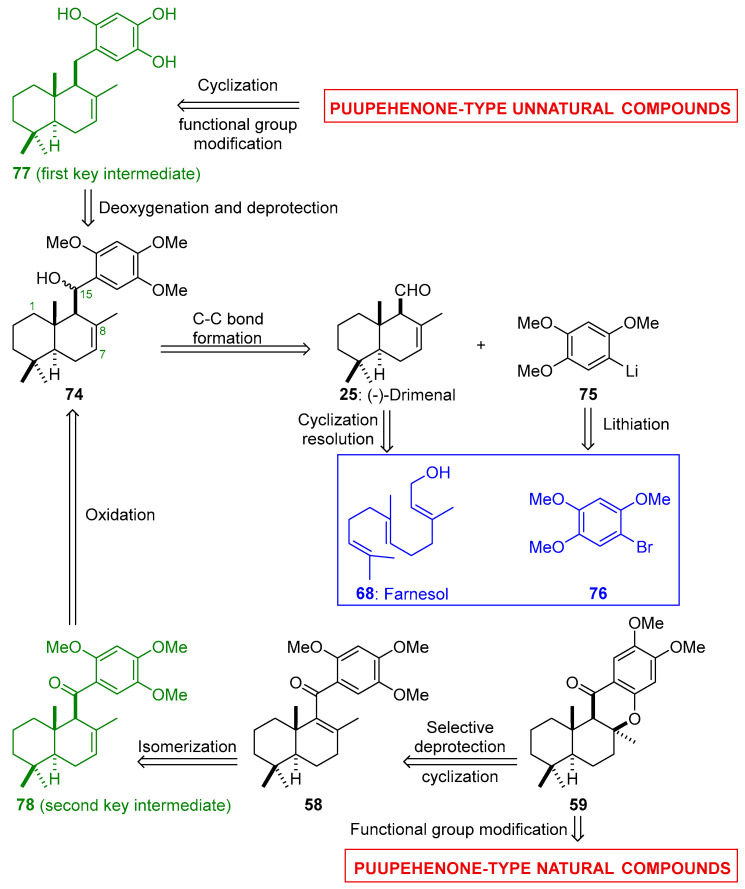
Unified retrosynthetic analysis of puupehenone-type natural and unnatural products.

In this section, based on the antecedents discussed, and in our personal experience in the total synthesis of marine natural products [22,45,46,47,48,49,50,51,52], we propose a retrosynthetic analysis towards a common intermediate **74** in which the carbon 15 is functionalized with a hydroxy group (Scheme 14). Our proposal should allow the preparation of either the natural puupehenone-type compound with total diastereoselectivity or, alternatively, their nonnatural epimers (C_8_-Me_β_) by previous hydrogenolysis of the hydroxy group on the carbon 15 of **74** (Scheme 14).

We consider that a convergent synthesis toward intermediate **74** is the best strategy for the synthesis of puupehenone-type products since it improves the efficiency of the multistep synthesis. Therefore, the key endocyclic compound **74** could be synthesized via a C–C bond coupling reaction between an electrophilic drimenal **25** with the aryllithium derivative **75**. Drimenal **25** can be obtained in optically pure form from *E*,*E*-farnesol by cationic cyclization followed by resolution of the racemic mixture via chromatographic separation of the diasteromeric camphanoate derivatives [53,54]. The aryllithium **75** would be formed by the lithiation of **76**. We propose the aromatic ring **76** as the second fragment in the coupling process with **25**, since it has been shown that it can be selectively demethylated [19], a step which is necessary for the completion of the synthetic route. Deoxygenation of the C15–OH group in **74** and subsequent total demethylation would afford the first key intermediate **77**. Acid-mediated cyclization of **77** would give 8-epi-puupehenol, from which puupehenone-type unnatural products (C_8_-Me_β_) would be obtained via the modification of its functional groups. On the other hand, for the synthesis of puupehenone-type natural compounds (C_8_-Me_α_), intermediate **74** should be oxidized, generating the ketone **78**, which would be the second key intermediate. The isomerization of the trisubstituted double bond in **78** would form the aryl vinyl ketone **58**. Selective demethylation and diastereoselective cyclization of **58** with KOH should lead to the tetracyclic compound **59** with the correct stereochemistry at C8, the one present in the puupehenone-type natural products, as we have previously commented [31]. Finally, there is a selection of different experimental conditions which could convert the tetracyclic compound **59** into puupehenones-type natural products. This general retrosynthetic analysis shown in Scheme 14 represents a unified, low-cost, and powerful tool for the construction of puupehenone-type natural products and their C8-epimers, both of which could be used to address relevant biological and pharmaceutical studies.

## 4. Conclusions

A review of the synthesis of marine puupehenone (**1**) and puupehedione (**2**) has been presented in terms of the starting material and synthetic strategy employed. Being complex structures, their synthesis often requires a reasonable number of synthetic operations. Taking into consideration the best results from each approach, we have described in this perspective article a new unified retrosynthetic analysis for the preparation of puupehenone-type natural and unnatural products. The proposal is designed to require the minimum synthetic operations and demonstrates its versatility and power for the preparation of these fascinating compounds, which could be used to address future biological and pharmaceutical issues.

## Data Availability

Data is contained within the article.

## References

[1] Marcos I.S., Conde A., Moro R.F., Basabe P., Diez D., Urones J.G. (2010). Quinone/Hydroquinone Sesquiterpenes. Mini-Rev. Org. Chem..

[2] Djura P., Stierle D.B., Sullivan B., Faulkner D.J., Arnold E., Clardy J. (1980). Some metabolites of the marine sponges Smenospongia aurea and Smenospongia (polyfibrospongia) echina. J. Org. Chem..

[3] Kohmoto S., McConnell O.J., Wright A. (1987). Puupehenone, a cytotoxic metabolite from a deep water marine sponge, Stronglyophora hartman. J. Nat. Prod..

[4] Pina I.C., Sanders M.L., Crews P. (2003). Puupehenone congeners from an indoPacific Hyrtiossponge. J. Nat. Prod..

[5] Faulkner D.J. (1998). Marine natural products. Nat. Prod. Rep..

[6] Hamann M.T., Scheuer P.J. (1991). Cyanopuupehenol, an antiviral metabolite of a sponge of the order Verongida. Tetrahedron Lett..

[7] Nasu S.S., Yeung B.K., Hamann M.T. (1995). Puupehenone-related metabolites from two Hawaiian sponges, *Hyrtios* spp.. J. Org. Chem..

[8] Navi B.N., Perzanovski H.P., Ross R.A., Erdman T.R., Scheuer P.J., Finar J., Clardy J. (1979). Recent research in marine natural products: The puupehenones. Pure Appl. Chem..

[9] Urban S., Capon R.J. (1996). Absolute Stereochemistry of Puupehenone and Related Metabolites. J. Nat. Prod..

[10] Martínez-Poveda B., Quesada A.R., Medina M.A. (2017). Pleiotropic Role of Puupehenones in Biomedical Research. Mar. Drugs.

[11] Barrero A.F., Álvarez-Manzaneda E.J., Chahboun R. (1997). Enantiospecific Synthesis of (+)-Puupehenone from (-)-Sclareol and Protocatechualdehyde. Tetrahedron Lett..

[12] Barrero A.F., Álvarez-Manzaneda E.E., Chahboun R., Cortés M., Armstrong V. (1999). Synthesis and Antitumor Activity of Puupehedione and related Compounds. Tetrahedron.

[13] Álvarez-Manzaneda E.J., Chahboun R., Barranco Pérez I., Cabrera E., Álvarez E., Álvarez-Manzaneda R. (2005). First Enantiospecific Synthesis of the Antitumor Marine Sponge Metabolite (−)-15-Oxopuupehenol from (−)-Sclareol. Org. Lett..

[14] Álvarez-Manzaneda E., Chahboun R., Cabrera E., Álvarez E., Haidour A., Ramos J.M., Álvarez-Manzaneda R., Tapia R., Es-Samti H., Fernández A. (2009). A Convenient Enantiospecific Route towards Bioactive Merosesquiterpenes by Cationic-Resin-Promoted Friedel–Crafts Alkylation with α,β-Enones. Eur. J. Org. Chem..

[15] Quideau S., Lebon M., Lamidey A.-M. (2002). Enantiospecific Synthesis of the Antituberculosis Marine Sponge Metabolite (+)-Puupehenone. The Arenol Oxidative Activation Route. Org. Lett..

[16] Wang H.-S., Li H.-J., Wang J.-L., Wu Y.-C. (2017). Protecting-group-free synthesis of haterumadienone- and puupehenone-type marine natural products. Green Chem..

[17] Wang H.-S., Li H.-J., Nan X., Luo Y.-Y., Wu Y.-C. (2017). Enantiospecific Semisynthesis of Puupehedione-Type Marine Natural Products. J. Org. Chem..

[18] Maiti S., Sengupta S., Giri C., Achari B., Banerjee A.K. (2001). Enantiospecific synthesis of 8-epipuupehedione from (R)-(−)-carvone. Tetrahedron Lett..

[19] Song H., Liu L., Yang M., Wu G., Chen P., Xie X., She X. (2020). Total syntheses of (−)-15-oxopuupehenol and (+)-puupehenone and formal syntheses of (−)-puupehenol and (+)-puupehedione. Org. Chem. Front..

[20] Armstrong V., Barrero A.F., Álvarez-Manzaneda J.E., Cortés M., Sepúlveda B. (2003). An Efficient Stereoselective Synthesis of Cytotoxic 8-Epipuupehedione. J. Nat. Prod..

[21] Trammell G.L. (1978). The total synthesis of (±)-puupehenone. Tetrahedron Lett..

[22] Gansäuer A., Justicia J., Rosales A., Worgull D., Rinker B., Cuerva J.M., Oltra J.E. (2006). Transition-Metal-Catalyzed Allylic Substitution and Titanocene-Catalyzed Epoxypolyene Cyclization as a Powerful Tool for the Preparation of Terpenoids. Eur. J. Org. Chem..

[23] Wu Y.-C., Cheng Y.-F., Li H.-J., Nandeshwarappa B.P. (2019). Approaches to the Total Synthesis of Puupehenone-Type Marine Natural Products. Organic Synthesis—A Nascent Relook.

[24] Wu Y.-B., Ni Z.-Y., Shi Q.-W., Dong M., Kiyota H., Gu Y.-C., Cong B. (2012). Constituents from *Salvia* Species and Their Biological Activities. Chem. Rev..

[25] Schalk M., Pastore L., Mirata M.A., Khim S., Schouwey M., Deguerry F., Pineda V., Rocci L., Daviet L. (2012). Toward a Biosynthetic Route to Sclareol and Amber Odorants. J. Am. Chem. Soc..

[26] Frija L.M.T., Frade R.F.M., Afonso C.A.M. (2011). Isolation, Chemical, and Biotransformation Routes of Labdane-type Diterpenes. Chem. Rev..

[27] Barrero A.F., Manzaneda E.A., Altajeros J., Salido S., Ramos J.M., Simmonds M.S.J., Blaney W.M. (1995). Synthesis of Biologically Active Drimanes and Homodrimanes from (-)-Sclareol. Tetrahedron.

[28] Barrero A.F., Álvarez-Manzanda J.E., Chahboun R. (1998). Synthesis of Wiedendiol-A and Wiedendiol-B from Labdane Diterpenes. Tetrahedron.

[29] Topcu G., Ulubelen A., Tam T.C.-M., Che C.-T. (1996). Norditerpenes and Norsesterterpenes from *Salvia yosgadensis*. J. Nat. Prod..

[30] Hajime H. (1971). Aroma of cigar tobacco. II. Isolation of norambreinolide from cigar tobacco. Agric. Biol. Chem..

[31] Dixon D., Lockner J.W., Zhou Q., Baran P.S. (2012). Scalable, Divergent Synthesis of Meroterpenoids via “Borono-sclareolide”. J. Am. Chem. Soc..

[32] Cheng Y., Lyu X., Liu C., Wang X., Cheng J., Zhang D., Meng X., Zhao Y. (2023). Synthesis and Biological Evaluation of Sclareolide-Indole Conjugates and Their Derivatives. Molecules.

[33] Ravid U., Bassat M., Putievsky E., Weistein V., Ikan R. (1987). Isolation and determination of optically pure carvone enantiomers from caraway (*Carum carvi* L.), dill (*Anethum graveolens* L.), spearmint (*Mentha spicata* L.) and *Mentha longifolia* (L.) Huds. Flavour Fragance J..

[34] Ncube E.N., Steenkamp L., Dubery I.A. (2020). Ambrafuran (Ambrox^TM^) Synthesis from Natural Plant Product Precursors. Molecules.

[35] Quideau S., Pouységu C., Deffieux D. (2008). Oxidative Dearomatization of Phenols: Why, How and What For?. Synlett.

[36] Noble A., Roesner S., Aggarwal V.K. (2016). Short Enantioselective Total Synthesis of Tatanan A and 3-*epi*-Tatanan A Using Assembly-Line Synthesis. Angew. Chem. Int. Ed..

[37] Appel H.H., Brooks C.J.W., Overto K.H. (1959). The Constitution and Stereochemistry of Drimenol, a Novel Bicyclic Xesquiterpenoid. J. Chem. Soc..

[38] Cortés M., Delgado V., Saitz C., Armstrong V. (2011). Drimenol: A Versatile Synthon for Compounds with trans-Drimane Skeleton. Nat. Prod. Commun..

[39] Chen X.X., Zhang D., Xu D., Zhou H., Xu G. (2020). Remote C−H Activation Strategy Enables Total Syntheses of Nortriterpenoids (±)-Walsucochin B and (±)-Walsucochinoids M and N. Org. Lett..

[40] Jung Y.-Y., Hwang S.T., Sethi G., Fan L., Arfuso F., Ahn K.S. (2018). Potential Anti-Inflammatory and Anti-Cancer Properties of Farnesol. Molecules.

[41] Rosales Martínez A., Pozo Morales L., Díaz Ojeda E., Castro Rodríguez M., Rodríguez-García I. (2021). The Proven Versatility of Cp_2_TiCl. J. Org. Chem..

[42] Ma T.-K., Parsons P.J., Barret A.G.M. (2019). Meroterpenoid Synthesis via Sequential Polyketide Aromatization and Radical Anion Cascade Triene Cyclization: Biomimetic Total Syntheses of Austalide Natural Products. J. Org. Chem..

[43] Gansäuer A., Rosales A., Justicia J. (2006). Catalytic epoxypolyene cyclization via radicals: Highly diastereoselective formal synthesis of puupehedione and 8-*epi*-puupehedione. Synlett.

[44] Snyder S.A., Corey E.J. (2006). Concise Total Syntheses of Palominol, Dolabellatrienone, β-Araneosene, and Isoedunol via an Enantioselective Diels−Alder Macrobicyclization. J. Am. Chem. Soc..

[45] Rosales A., López-Sánchez C., Álvarez-Corral M., Muñoz-Dorado M., Rodríguez-García I. (2007). Total Synthesis of (±)-Euryfuran Through Ti(III) Catalyzed Radical Cyclization. Lett. Org. Chem..

[46] Rosales A., Muñoz-Bascón J., Moráles-Álcazar V.M., Castilla-Alcalá J.A., Oltra J.E. (2012). Ti(III)-catalyzed, concise synthesis of marine furanospongian diterpenes. RSC Adv..

[47] Rosales A., Muñoz-Bascón J., López-Sánchez C., Álvarez-Corral M., Muñoz-Dorado M., Rodríguez-García I., Oltra J.E. (2012). Ti-Catalyzed Homolytic Opening of Ozonides: A sustainable C-C Bond-Forming Reaction. J. Org. Chem..

[48] Rosales A., Foley L.A.R., Padial N.M., Muñoz-Bascón J., Sancho-Sanz I., Roldán-Molina E., Pozo-Morales L., Irías-Álvarez A., Rodríguez-Maecker R., Rodríguez-García I. (2016). Diastereoselective Synthesis of (±)-ambroz by Titanium(III)-Catalyzed Radical Tandem Cyclization. Synlett.

[49] Rosales Martinez A., Pozo Morales L., Diaz Ojeda E. (2019). Cp_2_TiCl-catalyzed, concise synthetic approach to marine natural product (±)-cyclozonarone. Synth. Commun..

[50] Rosales Martínez A., Enríquez L., Jaraíz M., Pozo Morales L., Rodríguez-García I., Díaz Ojeda E. (2020). A Concise Route for the Synthesis of Tetracyclic Meroterpenoids: (±)-Aureol Preparation and Mechanistic Interpretation. Mar. Drugs.

[51] Torres-García I., López-Martínez J.L., López-Domene R., Muñoz-Dorado M., Rodríguez-García I., Álvarez-Corral M. (2022). Enantioselective total synthesis of putative dihydrorosefuran, a monoterpene with an unique 2,5-dihydrofuran structure. Beilstein J. Org. Chem..

[52] López-Martínez J., Torres-García I., Moreno-Gutiérrez I., Oña-Burgos P., Rosales Martínez A., Muñoz-Dorado M., Álvarez-Corral M., Rodríguez-García I. (2023). A Concise Diastereoselective Total Synthesis of α-Ambrinol. Mar. Drugs.

[53] Arjona O., Garranzo M., Mahugo J., Maroto E., Plumet J., Sáez B. (1997). Total synthesis of both enantiomers of 15-oxopuupehenol methylendioxy derivatives. Tetrahedron Lett..

[54] Jordine G., Bick S., Mgller U., Wezel P., Daucher B., Maas G. (1994). Studies on forskolin ring C forming reactions. Tetrahedron.

